# Clinical Presentation and Early Outcomes of Congenital Endocrine Salt‐Wasting Syndromes Unrelated to 21‐Hydroxylase Deficiency

**DOI:** 10.1002/edm2.70281

**Published:** 2026-07-19

**Authors:** Vittorio Ferrari, Valentina Assirelli, Emma Bonaguri, Giovanni Silva, Rita Ortolano, Federico Baronio, Marcello Lanari, Antonio Balsamo

**Affiliations:** ^1^ Pediatric Unit IRCCS Azienda Ospedaliero Universitaria di Bologna Bologna Italy; ^2^ Primary Care Pediatricians Azienda Unità Sanitaria Locale (AUSL) Imola Italy; ^3^ Specialty School of Pediatrics Alma Mater Studiorum, University of Bologna Bologna Italy; ^4^ Department of Medical and Surgical Sciences Alma Mater Studiorum, University of Bologna Bologna Italy

**Keywords:** adrenal insufficiency, aldosterone synthase deficiency, congenital adrenal hypoplasia, early‐onset salt‐wasting, growth outcomes, newborn screening, pseudohypoaldosteronism type 1

## Abstract

**Objective:**

Congenital endocrine salt‐wasting syndromes unrelated to 21‐hydroxylase deficiency are rare disorders with overlapping clinical and biochemical features at presentation. This study described the spectrum, early clinical course, and 36‐month outcomes of these conditions in the era of newborn screening, and assessed whether severity at presentation was associated with later treatment requirements and growth.

**Methods:**

Retrospective single‐center cohort study including infants diagnosed between 1989 and 2023 with endocrine salt‐wasting syndromes unrelated to 21‐hydroxylase deficiency. Clinical presentation, biochemical findings, treatment requirements, genetic data, and longitudinal growth outcomes up to 36 months were analysed.

**Results:**

Twenty patients were included: eight with aldosterone synthase deficiency, six with renal pseudohypoaldosteronism type 1, four with systemic pseudohypoaldosteronism type 1, and two with congenital adrenal hypoplasia. Systemic pseudohypoaldosteronism type 1 presented earliest and with the most severe biochemical abnormalities, requiring higher sodium supplementation at onset. Aldosterone synthase deficiency and renal pseudohypoaldosteronism type 1 presented later, with less severe, overlapping biochemical profiles. Differences in early management across etiologies were mainly limited to sodium supplementation, whereas time to electrolyte stabilization and mineralocorticoid initiation did not differ significantly. In exploratory analyses, severity at presentation was not associated with later treatment requirements or growth outcomes, whereas growth was largely preserved, with greater auxological vulnerability in systemic pseudohypoaldosteronism type 1.

**Conclusions:**

Congenital endocrine salt‐wasting syndromes unrelated to 21‐hydroxylase deficiency show substantial overlap at onset, whereas disease‐specific features become more recognizable during follow‐up. Growth outcomes were generally preserved with appropriate management and did not appear to be influenced by clinical severity at presentation.

## Graphical abstract

Early‐onset endocrine salt‐wasting syndromes unrelated to 21‐hydroxylase deficiency overlap markedly at presentation, limiting etiological distinction. In this 20‐patient cohort, disease‐specific patterns emerged during follow‐up, while growth was generally preserved and initial biochemical severity was not associated with later treatment burden or growth outcome.

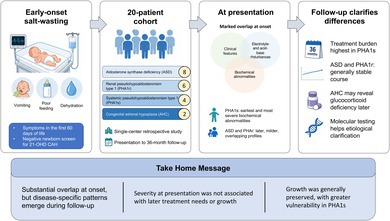

## 1 Introduction

Salt‐wasting (SW) is an important cause of hospitalization during the first weeks of life and a potentially life‐threatening condition if not promptly recognized and treated.

It results from impaired maintenance of sodium balance and is typically characterized by hyponatremia, with or without hyperkalemia, hypochloremia, and metabolic acidosis. Clinical manifestations are variable and often nonspecific, and may include vomiting, irritability, hypotonia, poor feeding, dehydration, poor weight gain, and, in extreme cases, seizures [1].

Newborns and young infants are particularly vulnerable to disturbances in sodium and water balance because of the physiological immaturity of renal and hormonal regulatory mechanisms. Reduced glomerular filtration rate, immaturity of the distal nephron, and partial transient renal resistance to aldosterone may limit sodium conservation and potassium homeostasis during early life, thereby increasing the susceptibility to clinically relevant SW when additional pathological conditions are present [2]. Extrarenal fluid losses, particularly those related to gastrointestinal conditions, are also well‐recognized contributors to sodium depletion and SW presentations in this age group [3].

SW may also occur in the context of congenital disorders. In this setting, SW represents a common manifestation of congenital adrenal insufficiency (CAI), with 21‐hydroxylase deficiency congenital adrenal hyperplasia (21OHD CAH) representing the most frequent endocrine cause [4]. However, endocrine causes of early‐life SW extend beyond 21OHD CAH and include a heterogeneous group of rarer disorders (Table 1). Although individually rare, these endocrine disorders are clinically relevant because they often present in the neonatal period or early infancy with symptoms and biochemical abnormalities that overlap with each other and with 21OHD CAH, making early etiological differentiation challenging. Endocrine causes of congenital SW syndromes unrelated to 21‐hydroxylase deficiency (Non‐21OHD SW) include aldosterone synthase deficiency (ASD), adrenal hypoplasia congenita (AHC), and renal and systemic forms of pseudohypoaldosteronism type 1 (PHA1r and PHA1s) [5, 6, 7, 8, 9]. Despite the clinical relevance and potential severity of these conditions, data on their incidence, prevalence, and clinical spectrum remain limited. The exact frequency of endocrine causes of early‐onset Non‐21OHD SW is unknown. In regions where neonatal screening for 21OHD CAH is available, including Emilia‐Romagna, where screening has supported the early diagnosis of classic 21OHD CAH since 1980 [10], the most common genetic cause of SW can be promptly excluded; however, in neonates or young infants presenting with SW and negative screening results, identification of an alternative endocrine aetiology is required to guide further diagnostic evaluation and management. However, in infants with Non‐21OHD SW, the clinical condition may be severe, and prompt identification of the underlying cause is essential for appropriate management. While clinical presentation and initial biochemical findings may orient the diagnostic workup, the substantial clinical and biochemical overlap among endocrine causes of congenital SW not detected by screening programs often limits diagnostic certainty in the acute phase. For this reason, definitive diagnosis frequently relies on molecular genetic testing.

**TABLE 1:** Etiological classification of congenital adrenal insufficiency.

Congenital adrenal hyperplasia	Adrenal hypoplasia congenita	Familial glucocorticoid deficiency	Metabolic causes	Glucocorticoid resistance	Mineralocorticoid resistance	Mineralocorticoid deficiency
Steroidogenic acute regulatory protein deficiency Cholesterol side‐chain cleavage deficiency 3β‐hydroxysteroid dehydrogenase deficiency 21‐hydroxylase deficiency 11β‐hydroxylase deficiency 17α‐hydroxylase/17,20‐lyase deficiency	NR0B1 gene mutation NR5A1 gene mutation IMAGE syndrome SERKAL syndrome	Familial glucocorticoid deficiency type 1 Familial glucocorticoid deficiency type 2 Familial glucocorticoid deficiency type 3 Nicotinamide nucleotide transhydrogenase deficiency Natural killer cell and glucocorticoid deficiency with DNA repair defect Triple A syndrome	Smith‐Lemli‐Opitz syndrome Adrenoleukodystrophy and adrenomyeloneuropathy Primary xanthomatosis Mitochondrial disorders	NR3C1 gene mutations	NR3C2 gene mutations	Corticosterone methyl oxidase deficiency type I Corticosterone methyl oxidase deficiency type II

*Note:* Conditions are grouped according to their main pathogenic mechanism, including defects of steroidogenesis (congenital adrenal hyperplasia), disorders of adrenal development (including adrenal hypoplasia congenita), familial glucocorticoid deficiency, metabolic disorders with adrenal involvement, primary glucocorticoid or mineralocorticoid resistance, and isolated mineralocorticoid deficiencies.

The primary aim of this study was to describe the spectrum and relative distribution of endocrine causes of early‐onset Non‐21OHD SW in a single‐center cohort. Secondary aims were: (i) to characterize and compare the clinical presentation, biochemical features, and genetic background associated with different etiologies; (ii) to highlight early diagnostic uncertainty in early‐onset SW and its implications for follow‐up and management; (iii) to assess therapeutic strategies and short‐ to medium‐term clinical outcomes, including biochemical control, treatment burden, and auxological trajectories.

The 36‐month longitudinal characterization of these rare conditions provides descriptive data on their early clinical course and may contribute to a better understanding of diagnostic uncertainty and disease‐specific management needs beyond the acute presentation.

## 2 Materials and Methods

### 2.1 Study Design and Population

This was a retrospective single‐center observational cohort study conducted at the Paediatric Endocrinology Unit of IRCCS Azienda Ospedaliero Universitaria di Bologna, Policlinico di Sant'Orsola. The study included all patients with early‐onset SW due to an endocrine cause other than 21OHD CAH treated in our Center between 1 January 1989 and 31 December 2023.

Cases were identified through review of clinical records of patients followed for endocrine SW disorders other than 21OHD CAH. Early onset was defined as clinical presentation within the first 60 days of life, to capture disorders presenting during the neonatal period or very early infancy, when clinical features and are comparable, while excluding later‐onset or milder presentations during infancy that were outside the scope of the present study.

Inclusion criteria were: (i) diagnosis of SW syndrome defined by laboratory‐confirmed hyponatremia (serum sodium < 130 mEq/L) attributable to Non‐21OHD SW; and (ii) clinical onset within the first 60 days of life.

Exclusion criteria included non‐endocrine causes of SW, 21OHD CAH, and clinical onset beyond 60 days of life.

### 2.2 Data Collection

Data were retrospectively extracted from clinical records. Variables collected at presentation included gestational age, birth weight, age at symptom onset, body weight, clinical features, family history, serum electrolytes, acid–base status when available, and hormonal parameters relevant to adrenal function, including plasma renin, aldosterone, adrenocorticotropic hormone (ACTH), and cortisol. Follow‐up data included serial auxological and biochemical assessments up to the last available visit, with a maximum follow‐up of 36 months. Weight and length/height standard deviation scores were calculated using World Health Organization growth standards for children younger than 5 years [11]. Because renin assays changed over time, follow‐up analyses were restricted to active renin concentration measurements.

Genetic data were collected when available from the retrieved clinical records. Molecular analyses had been performed as part of routine clinical care using a candidate gene approach based on clinical and biochemical suspicion, with direct Sanger sequencing of genes selected according to the most likely diagnosis, including *CYP11B2*, *NR0B1*, *NR3C2*, and *SCNN1B*. Variants are reported according to Human Genome Variation Society nomenclature and were interpreted according to standard clinical laboratory criteria in use at the time of analysis. For some older cases, documented molecular confirmation was not available in the retrieved records. These patients were retained in the cohort when the diagnosis reported in the available clinical records was consistent with the clinical, biochemical, hormonal, therapeutic, and longitudinal data reviewed for this study.

### 2.3 Statistical Analysis

Continuous variables were assessed for normality using the Shapiro–Wilk test and are reported as mean ± standard deviation (SD) or median and interquartile range (IQR), as appropriate. For variables subject to right‐censoring due to assay upper limits (e.g., plasma renin), censored observations were treated as right‐censored for median calculation and assigned the upper limit of quantification for estimation of the minimum possible mean. Comparisons across diagnostic groups were performed using the Kruskal‐Wallis or Mann–Whitney U test, as appropriate. Correlations were assessed using Spearman's rank coefficient. Longitudinal changes were evaluated descriptively because of the limited sample size. Relative variability of mineralocorticoid and sodium chloride dosages over time was assessed using the coefficient of variation (CV%). A two‐sided *p* value < 0.05 was considered statistically significant. Analyses were performed using R version 4.4.2 (R Foundation for Statistical Computing, Vienna, Austria).

### 2.4 Ethics Statement

The study was conducted in accordance with the Declaration of Helsinki and approved by the Ethics Committee AVEC (Area Vasta Emilia Centro; 23/2025/Oss/AOUBO, 22 January 2025). Written informed consent for study participation and data processing was obtained from parents or legal guardians of patients in active follow‐up. For retrospective data from untraceable patients, data processing without consent was permitted under the approved protocol and applicable Italian regulations.

## 3 Results

### 3.1 Salt Wasting Syndrome Onset

A total of 20 patients with early‐onset Non‐21OHD SW were included in the analysis. According to the underlying aetiology, patients were classified into four diagnostic groups: ASD (*n* = 8), AHC (*n* = 2), PHA1r (*n* = 6), and PHA1s (*n* = 4).

Documented molecular confirmation was available in 13 patients and unavailable in the retrieved records for 7 older cases. In these cases, the diagnosis reported in the available clinical records was retained after retrospective review of the clinical, biochemical, hormonal, therapeutic, and longitudinal data.

Table 2 reports individual genetic, clinical, biochemical, and treatment data at disease onset, whereas Table 3 summarizes mean biochemical findings and early treatment course by disease group.

**TABLE 2:** Genetic, clinical, biochemical, and therapeutic characteristics at disease onset in the study cohort.

Diagnosis	Pt	Genetic Findings		Clinical Presentation						Biochemical Profile				Early Management			
		Gene	Pathogenic Variant *	Onset age, d	Vomiting	Poor weight gain	Dehydration	Hypotonia	Feeding difficulties	Sodium (mmol/L)	Potassium (mmol/L)	Renin (ng/mL/h^▲^; μU/mL^●^)	Aldosterone (ng/dL)	Therapy	MC start, d	NaCl start, d	Time to balance, d
ASD	*1*	*CYP11B2*	c.1066C>T; c.1151G>A [p.Arg356Trp; p.Gly384Asp]	41	√	√			√	124	6,7	1340^●^	10,1	MC + NaCl	45	41	5
	*2*	*CYP11B2*	c.422G>A; c.1393_1395delTGC [p.Arg141Gln; p.Val465del]	12		√			√	121	7,4	998^●^	3,6	MC + NaCl	18	18	4
	*3*	*CYP11B2*	c.390C>A; c.763G>T [p.Tyr130Ter; p.Gly255Val]	47	√	√	√	√		125	7,1	> 500^●^	2,66	MC + NaCl	61	58	10
	*4*	*CYP11B2*	c.1066C>T; c.1151G>A [p.Arg356Trp; p.Gly384Asp]	5		√			√	123	10,9	516,7^●^	3	MC + NaCl	6	8	6
	*5*	*CYP11B2*	c.520_525delCGGCGG;c.788T>A;c.1156T>C [p.Arg174_Arg175del; p.Ile236Asn; p.Val386Ala]	16	√	√	√			122	8	> 500^▲^	5,4	MC + NaCl	22	18	35
	*6*	*CYP11B2*	c.395+1G>T (homozygous) [p.? splice‐site variant]	28	√	√	√			128	7	> 500^▲^	10	MC + NaCl	35	30	45
	*7*	*CYP11B2*	c.421C>T; c.593A>G [p.Arg141Ter; p.Asn198Ser]	19		√		√	√	116	8,4	> 500^▲^	14	MC + NaCl	27	19	38
	*8*	*CYP11B2*	c.788T>A (homozygous) [p.Ile263Asn]	10		√			√	127	6,4	1424^●^	8,6	MC + NaCl	23	10	13
AHC	*9*	—	—	15		√		√		128	8,2	915^▲^	14,1	MC + NaCl	15	15	6
	*10*	*NR0B1*	c.1288_1290delAAC [p.Asn430del]	10	√	√	√			118,6	6,7	> 500^▲^	5,6	*NA*	*NA*	*NA*	*NA*
PHA1r	*11*	*NR3C2*	c.2309C>G (heterozygous) [p.Ser770Cys]	27		√			√	131	6,7	729^●^	282,6	NaCl	—	28	5
	*12*	*NR3C2*	c.2657T>G (heterozygous) [p.Leu886Arg]	11	√	√				127	5,8	359,1^▲^	417	NaCl	—	14	19
	*13*	*NR3C2*	c.2125delA (heterozygous) [p.Leu709CysfsTer3]	21	√	√				120	7,3	> 500^▲^	300	NaCl	—	23	25
	*14*	—	—	56	√	√				118	6,5	> 500^▲^	300	*NA*	*NA*	*NA*	*NA*
	*15*	*NR3C2*	c.402T>A (heterozygous) [p.Tyr134Ter]	18		√				126	5,4	306^●^	862	NaCl	—	18	6
	*16*	—	—	24		√				124	6,1	> 500^▲^	585,6	*NA*	*NA*	*NA*	*NA*
PHA1s	*17*	—	—	5	√	√	√			125	11	> 500^▲^	3048	*NA*	*NA*	*NA*	*NA*
	*18*	*SCNN1B*	c.521T>A (homozygous) [p.Leu174Ter]	8		√		√		132	9,8	> 500^▲^	4000	MC + NaCl	14	8	21
	*19*	—	—	2		√		√	√	126	6	9340^●^	1200	MC + NaCl	31	2	4
	*20*	—	—	7	√				√	126	11	1122^▲^	3784	*NA*	*NA*	*NA*	*NA*

*Note:* By presenting individual data, the table shows the heterogeneity of early presentation and the degree of clinical and biochemical overlap among diagnoses. Genetic variants are described according to HGVS nomenclature using NM_000498.3 for *CYP11B2*, NM_000475.6 for *NR0B1*, NM_000901.5 for *NR3C2*, and NM_001038.5 for *SCNN1B*. For splice‐site variants with undetermined protein consequences, the protein effect is reported as p.?. Check marks (√) indicate the presence of the corresponding clinical feature at disease onset. Renin values reported as > 500 indicate concentrations above the upper analytical limit. Reference ranges: sodium, 135–145 mmol/L; potassium, 3.5–5.5 mmol/L; plasma renin activity (▲), 0.2–2.2 ng/mL/h; plasma renin concentration (●), 4.2–59.7 μU/mL; aldosterone, 5–30 ng/dL.

Abbreviations: *−*, not available in the retrieved records; AHC, adrenal hypoplasia congenita; ASD, aldosterone synthase deficiency; d, days; NA, data not available; PHA1r, renal pseudohypoaldosteronism type 1; PHA1s, systemic pseudohypoaldosteronism type 1. Pt, patient number.

**TABLE 3:** Neonatal characteristics, biochemical findings at disease onset, and early treatment parameters, stratified by disease group.

	Neonatal characteristics		At disease onset										Treatment initiation age		Treatment average dose		
	GA (weeks)	BW (g)	Weight (g)	Age (days)^§^	Na (mmol/L)	K (mmol/L)^§^	Cl (mmol/L)	PRA (ng/mL/h)	PRC (μU/mL)	Aldosterone (ng/dL)	ACTH (pg/mL)	Cortisol (ng/mL)	MC (days)	NaCl (days)	MC (mg/day)	NaCl (mEq/day)^§^	Time to electrolytes balance (days)
ASD	39.8 ± 1.3	3025.6 ± 321.9	2965.7 ± 331.2	22.2 ± 5.1	123.2 ± 3.8	7.7 ± 1.4	93.5 ± 1.3	> 500^†^	955.7 ± 438.5*	7.17 ± 4.12	17.53 ± 15.92	42.35 ± 36.32	29.6 ± 17.1	25.2 ± 16.9	0.0875 ± 0.01	9.4 ± 2.9	19.5 ± 16.9
AHC	39^‡^	3525 ± 91.9	3716^‡^	12.5 ± 3.5	123.3 ± 6.6	7.4 ± 1.1	96^‡^	707.5 ± 293.4^#^	^#^	9.85 ± 6.01	368.5 ± 89.8	92.0 ± 80.6	15^‡^	15^‡^	0.1^‡^	48.0^‡^	6^‡^
PHA1r	37.25 ± 2.9	2876.7 ± 560.9	2806.3 ± 570.2	26.2 ± 15.6	124.3 ± 4.8	6.3 ± 0.7	94.0 ± 4.2	464.8 ± 70.4*	517.5 ± 299.1	457.9 ± 228.8	12.4 ± 6.11	104.25 ± 61.57	**	20.7 ± 6.1	**	5.7 ± 2.1	13.7 ± 9.8
PHA1s	37.0 ± 2.8	3065 ± 475.9	2547 ± 584.1	5.5 ± 2.6	127.25 ± 3.2	9.33 ± 2.9	102^‡^	707.3 ± 359.1*	9340^‡^	3008 ± 1272.4	15.67 ± 8.14	113.0 ± 69.63	22.5 ± 12.0	5.0 ± 4.2	0.15 ± 0.07	36 ± 16.9	12.5 ± 12.02

*Note:* Group summaries show how early biochemical abnormalities and initial treatment requirements were distributed across etiologies, providing an overview of the patterns observed during acute presentation and stabilization. Values are presented as mean ± SD unless otherwise specified. *Note:* Reference ranges: sodium, 135–145 mmol/L; potassium, 3.5–5.5 mmol/L; chloride, 98–106 mmol/L; plasma renin activity, 0.2–2.2 ng/mL/h; plasma renin concentration, 4.2–59.7 μU/mL; aldosterone, 5–30 ng/dL; ACTH, 10–60 pg/mL; cortisol, 50–250 ng/mL.

Abbreviations: ACTH, adrenocorticotropic hormone; BW, birth weight; Cl, chloride; GA, gestational age; K, potassium; MC, mineralocorticoid therapy; Na, sodium; NaCl, oral sodium supplementation.PRA, plasma renin activity; PRC, plasma renin concentration.

* Censored renin values exceeding the upper analytical limit (> 500) were assigned the upper limit value to calculate minimum possible.

^‡^ Values reported as > 500.

** indicates that the treatment was not administered.

^#^ indicates that no data were available.

^§^ indicates statistically significant differences across disease groups (Kruskal‐Wallis test): age at onset (*p* = 0.02), potassium (*p* = 0.04), and NaCl dose (*p* = 0.02). AHC was excluded from statistical comparisons because of the small sample size.

These data are reported to document the clinical spectrum at presentation, the extent of biochemical overlap among etiologies, and the range of early therapeutic management observed in routine clinical practice.

A comparative analysis across etiologies was performed excluding patients with AHC from formal statistical testing due to the small sample size. Given the limited number of patients in each group, all statistical comparisons should be considered exploratory and mainly descriptive. Age at onset differed significantly among ASD, PHA1r, and PHA1s (Kruskal‐Wallis test, *p* = 0.02), with PHA1s showing the earliest presentation. Potassium levels at presentation also differed significantly across groups (*p* = 0.04), with higher values observed in the PHA1s cases in this cohort. In contrast, sodium concentrations at onset showed substantial overlap and did not significantly differ among etiologies (*p* = 0.31). Initial sodium chloride supplementation requirements varied significantly across diagnostic groups (*p* = 0.02), with higher doses required in patients with PHA1s. By contrast, the age at initiation of sodium supplementation did not differ significantly among groups (*p* = 0.10). Furthermore, neither the initial dose of mineralocorticoid supplementation (*p* = 0.10) nor the age at initiation of mineralocorticoid therapy (*p* = 0.60) differed significantly across groups. Similarly, the time required to achieve electrolyte equilibrium after treatment initiation showed wide interindividual variability and did not differ significantly among etiologies (*p* = 0.77).

### 3.2 Salt Wasting Syndrome Follow‐Up

Table 4 reports auxological data, biochemical findings, and treatment requirements at 36 months of follow‐up, stratified by disease group.

**TABLE 4:** Auxological data, biochemical findings, and treatment requirements at 36 months of follow‐up, stratified by disease group.

	Number of patients	At 36‐month follow‐up										
		Height (cm)	Height (SDS)	Weight (kg)	Weight (SDS)	Na (mmol/L)	K (mmol/L)	PRC (μU/mL)	Aldosterone (ng/dL)	MC (μg/kg)	GC (mg/kg)	NaCl (mEq/kg)^§^
ASD	4	90.6 [83.9–95.6]	−1.30 [−2.20 to −0.35]	12.7 [10.6–14.3]	−0.53 [−2.16 to −0.16]	136.5 [135–139.5]	4.4 [4.3–4.7]	65.9 [48.2–98.4]	*NA*	6 [5–7] (*n* = 4)	*	1.39 [0.60–2.17] (*n* = 2)
AHC	1	95.7	−0.02	14.6	0.19	140	4.1	14.8	3.6	5	0.68	^#^
PHA1r	4	94.3 [92.1–96.0]	−0.45 [−0.74 to 0.10]	13.9 [13.2–14.8]	−0.22 [−0.55 to 0.20]	140 [137.5–141]	4.7 [4.4–4.8]	139.5 [61.7–217.2]	111.95 [56.15–395.95]	*	*	2.09 [1.38–4.12] (*n* = 4)
PHA1s	1	89.5	−2.00	11.7	−1.83	134	4.0	20.0	*NA*	*	*	4.78 (*n* = 1)

*Note:* These data summarize biochemical control, growth status, and ongoing treatment requirements at 36 months, showing the short‐term clinical status reached after early management. Auxological, biochemical, and treatment variables are reported as median [IQR], unless only one observation was available, in which case the single value is shown. For treatment variables, the number of patients receiving active treatment at 36 months is indicated in parentheses.

Abbreviations: GC, glucocorticoid therapy; MC, mineralocorticoid therapy; *NA*, not available; NaCl, oral sodium supplementation; PRC, plasma renin concentration.

^#^ Treatment discontinuation.

* The treatment was not administered.

^§^ indicates a statistically significant difference between ASD and PHA1r at 36 months for NaCl supplementation (Mann–Whitney U test, *p* = 0.02).

Exploratory analyses of the relationship between biochemical severity at disease onset and both treatment requirements and auxological outcomes at follow‐up were restricted to patients with ASD and PHA1r, the only groups with comparable longitudinal data. Eight patients (ASD, *n* = 4; PHA1r, *n* = 4) had follow‐up extending to 36 months of age and were therefore included in the combined ASD/PHA1r cohort (*n* = 8) for correlation analyses. In the combined cohort, sodium concentrations at onset were not significantly associated with auxological outcomes at 36 months (height SDS: ρ = −0.07, *p* = 0.86; weight SDS: ρ = −0.19, *p* = 0.65). Similarly, potassium levels at onset were not significantly associated with height SDS (ρ = −0.32, *p* = 0.43) or weight SDS (ρ = −0.11, *p* = 0.79) at 36 months. No significant associations were observed between biochemical severity at onset and NaCl supplementation at 36 months, either for sodium concentrations at onset (ρ = 0.07, *p* = 0.86) or potassium levels (ρ = −0.48, *p* = 0.19). Furthermore, the time required to achieve electrolyte equilibrium after treatment initiation was not significantly associated with auxological outcomes at 36 months (height SDS: ρ = −0.40, *p* = 0.60; weight SDS: ρ = −0.20, *p* = 0.80). Despite the absence of significant correlations between biochemical severity at onset and NaCl supplementation at follow‐up, NaCl requirements at 36 months differed significantly between diagnostic groups. Median NaCl dose was 1.39 mEq/kg (0.60–2.17) in ASD and 2.09 mEq/kg (1.38–4.12) in PHA1r (Mann–Whitney U test, *p* = 0.02).

**FIGURE 1:**
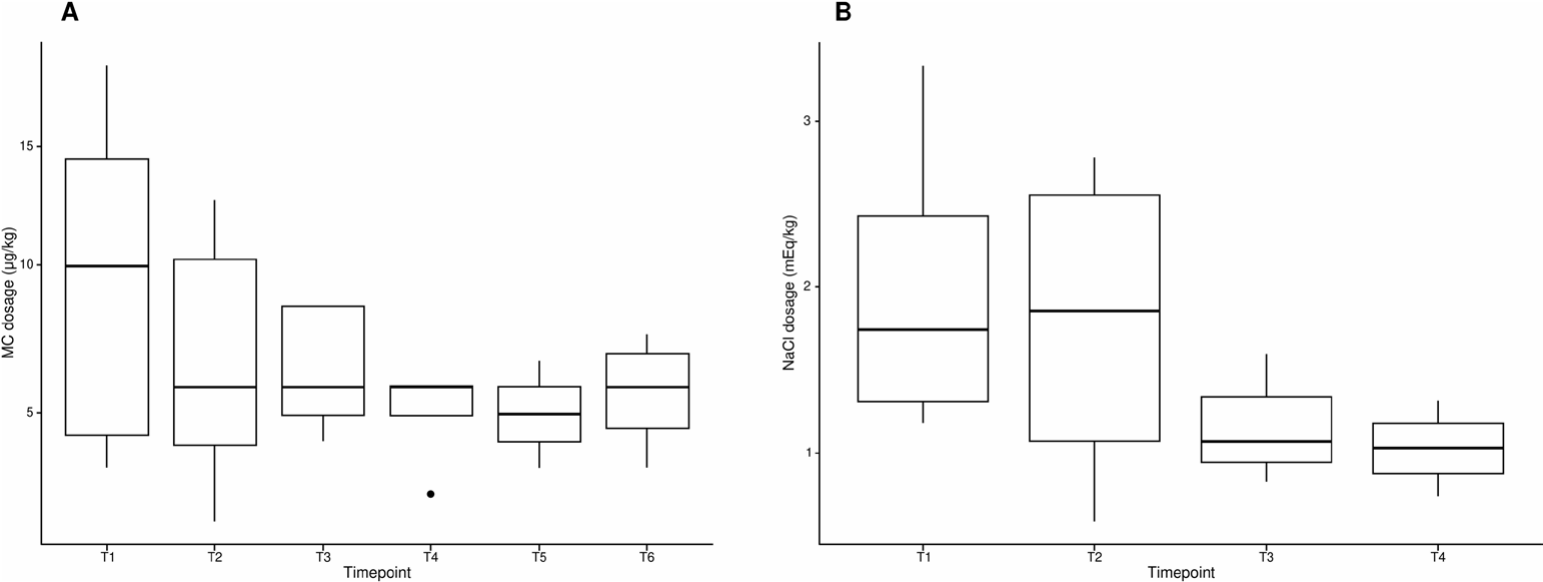
Boxplots of mineralocorticoid (MC, μg/kg) dosages and sodium chloride (NaCl, mEq/kg) supplementation in patients with aldosterone synthase deficiency (ASD) during follow‐up (A) MC dosages at each follow‐up timepoint (T1–T6). (B) NaCl supplementation at each follow‐up timepoint (T1–T4). Scheduled follow‐up timepoints correspond to 6, 12, 18, 24, 30, and 36 months after diagnosis. NaCl supplementation is shown up to T4 only. Boxplots represent the median, interquartile range, and distribution of individual dosages.

In patients with ASD, no significant correlations were observed between biochemical severity at onset and MC dose at 36 months, either for sodium concentrations at onset (ρ = 0.30, *p* = 0.62) or potassium levels (ρ = −0.10, *p* = 0.87). In the same group, the distribution of MC and NaCl dosages over follow‐up is shown in Figure 1. Relative interindividual variability in MC dosage, expressed as CV%, was higher at early timepoints (T1: 63%; T2: 69%) and lower at subsequent assessments (T3–T6: 34%–40%). The number of observations decreased over follow‐up because of treatment discontinuation or unavailable data. Variability in NaCl supplementation was evaluated over the first four timepoints only (T1–T4), as treatment was progressively discontinued in several patients and dosage data at later timepoints were frequently unavailable. The CV% for NaCl dosing was 48% at T1, 57% at T2, 33% at T3, and 39% at T4.

**FIGURE 2:**
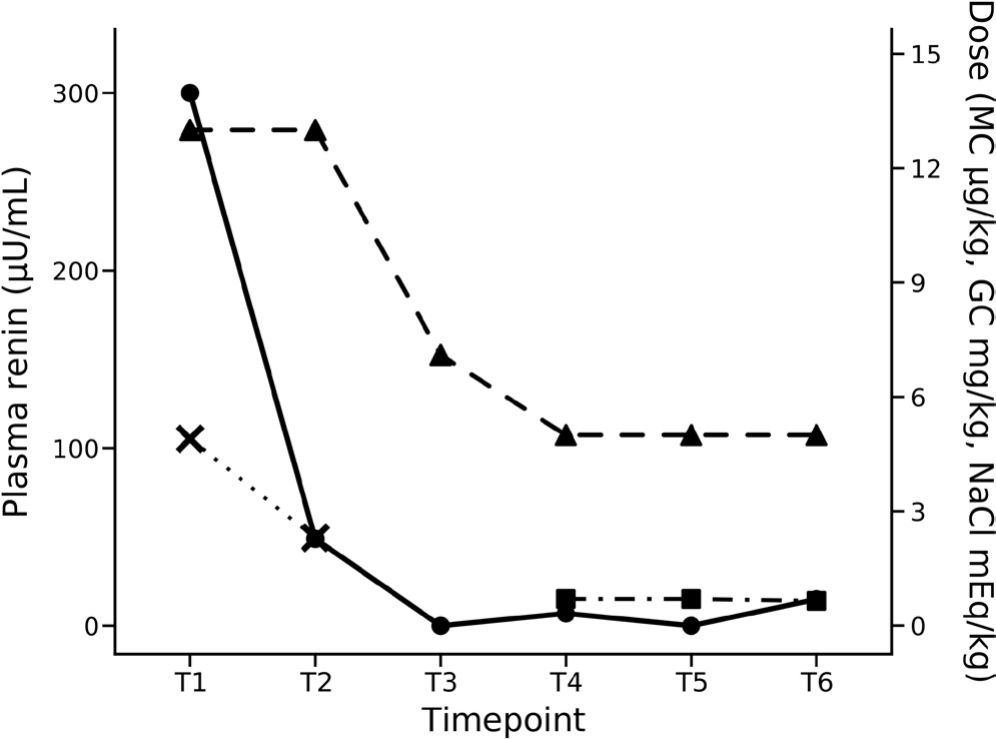
Longitudinal trajectories of plasma renin concentrations and replacement therapy dosing in the patient with adrenal hypoplasia congenita (AHC) during follow‐up. Plasma renin concentrations (μU/mL; left Y‐axis; solid line) are shown alongside daily dosages of mineralocorticoid (MC, μg/kg; dashed line), glucocorticoid (GC, mg/kg; dot–dash line), and sodium chloride (NaCl, mEq/kg; dotted line) displayed on the right Y‐axis. Measurements were obtained at scheduled follow‐up timepoints (T1–T6), corresponding to 6, 12, 18, 24, 30, and 36 months after diagnosis. Missing data points reflect unavailable measurements or periods before initiation or after discontinuation of specific therapies.

Only one patient with AHC completed the 36*–*month follow‐up; therefore, for this group, only a graphical representation of longitudinal plasma renin concentrations and replacement therapy dosing is provided (Figure 2).

## 4 Discussion

In this single‐center cohort, ASD was the most frequent endocrine cause of early‐onset Non‐21OHD SW, followed by renal and systemic PHA1 and AHC. Although early clinical management was broadly similar across etiologies, longitudinal follow‐up revealed differences in ongoing treatment needs and sodium supplementation requirements. Overall, in exploratory analyses, biochemical severity at presentation was not associated with treatment burden or auxological outcomes, and growth during the first 36 months of life was generally preserved.

### 4.1 Early Clinical Heterogeneity in Non‐21OHD SW

In this cohort, early‐onset Non‐21OHD SW showed both overlapping features at presentation and clinically relevant differences across etiologies. Patients with PHA1s presented earlier and had higher potassium levels and higher initial sodium supplementation requirement, consistent with the severe neonatal phenotype described in ENaC‐related disease [12, 13]. By contrast, patients with ASD and PHA1r showed a later onset and largely overlapping early profiles, with lower sodium supplementation requirements at presentation. Given the rarity of the individual disorders and the small number of cases, these findings should be interpreted as descriptive cohort observations; moreover, early biochemical values and treatment requirements may also be influenced by timing and frequency of monitoring and dose‐adjustment practices during acute management. Notably, differences in acute management were otherwise limited, and neither timing nor dose of MC initiation nor time to electrolyte stabilization differed significantly among patients receiving MC replacement. These findings suggest that early clinical and biochemical markers may be insufficient during the acute early‐life presentation to reliably distinguish among rare causes of salt‐wasting, particularly when hormonal assessment is incomplete or performed after treatment initiation, as often occurs in routine clinical practice [14, 15, 16].

In the extended follow‐up analysis, the severity of electrolyte imbalance at presentation was not associated with auxological outcomes or treatment intensity. Growth remained largely preserved during the first 36 months of life across the evaluated groups, consistent with previous reports in ASD showing rapid catch‐up growth and favourable auxological outcomes once appropriate MC and sodium replacement is established [17]. These findings suggest that early biochemical severity did not appear to translate into greater treatment burden or poorer auxological outcome during the first 36 months of life. Short‐term clinical evolution appeared to reflect longitudinal disease course and individualized management rather than presentation severity alone.

### 4.2 Longitudinal Course During Follow‐Up

Despite the partial overlap observed at presentation, longitudinal follow‐up highlighted differences in treatment needs and short‐term clinical course across etiologies. In ASD, severe early‐life biochemical abnormalities were followed by a generally favourable clinical course under treatment but with marked interindividual variability in MC and NaCl supplementation requirements over time. This observation is consistent with previous reports describing ASD as a condition with severe early‐life presentation but progressive clinical stabilization during childhood when adequately treated [5, 17, 18]. Renin concentrations may remain elevated despite clinical stability, as previously reported [17]. Similarly, patients with PHA1r showed stable sodium and potassium levels, largely normal growth trajectories, and a progressive reduction or discontinuation of NaCl supplementation over time, despite persistently increased renin and aldosterone concentrations. Persistent renin and aldosterone elevation despite clinical stability has also been described in PHA1r [13, 19, 20].

A different longitudinal pattern emerged in AHC and PHA1s. In AHC, although only one patient had complete longitudinal follow‐up, the early course was dominated by MC deficiency, while GC insufficiency became evident only later. This temporal evolution is consistent with previous reports indicating that adrenal insufficiency in AHC may evolve over time [6, 21, 22, 23]. In our patient, this temporal sequence contributed to a preliminary suspicion of ASD rather than AHC, highlighting the need for repeated diagnostic reassessment when SW is the predominant feature at presentation. By contrast, PHA1s was associated with the greatest treatment burden, including very high sodium requirements and greater auxological vulnerability, in line with previous descriptions of systemic PHA1 [12, 13].

These patterns indicate that clinically informative differences emerged over time and that follow‐up was more informative than initial presentation in defining each clinical trajectory.

### 4.3 Diagnostic Considerations in the Era of Newborn Screening

The implementation of newborn screening for 21OHD CAH has substantially improved early diagnosis and survival in affected patients. In settings where newborn screening for 21OHD CAH is available, Non‐21OHD etiologies have become more relevant in the differential diagnosis of early‐onset SW. Previous reports have emphasized that a negative newborn screening result for CAH may inadvertently delay further endocrine evaluation in symptomatic neonates [24]. This issue is particularly relevant in the early neonatal period, when electrolyte abnormalities may be the dominant manifestation and hormonal profiles may still be incomplete or evolving. Therefore, a negative newborn screening result for 21OHD CAH does not exclude other endocrine causes of early‐onset SW, which may still present with severe electrolyte imbalance. In this context, careful clinical assessment and targeted hormonal evaluation remain important in symptomatic infants, regardless of newborn screening results. Isolated biochemical parameters or single timepoint measurements may be difficult to interpret, particularly when samples are obtained after empiric steroid treatment or during phases of partial compensation [15]. In these cases, serial evaluations and longitudinal interpretation of hormonal patterns may be needed to clarify the diagnosis.

### 4.4 Molecular Analysis as a Key Step in Etiological Clarification

Genetic testing represents an increasingly important component of this diagnostic pathway. In patients with early‐onset Non‐21OHD SW, the marked overlap in clinical and biochemical features at onset across different etiologies often provides limited guidance for selecting the most appropriate gene for initial analysis. In this setting, targeted next‐generation sequencing (NGS) panels could allow more comprehensive and timely etiological clarification and support earlier treatment decisions, counselling, and long‐term follow‐up. In our cohort, available molecular analyses were performed through targeted single‐gene testing, reflecting the diagnostic approach used at the time; in similar cases, an NGS panel approach could allow more timely etiological clarification. Moreover, patients with the same underlying diagnosis showed differences in age at onset and initial clinical presentation. Although our study was not designed to explore genotype–phenotype correlations, this variability suggests that genetic factors may contribute to differences in disease expression. In this context, earlier molecular diagnosis may be relevant not only for timely etiological clarification, but also for identifying specific genotype features associated with differences in clinical course within the same disorder.

### 4.5 Strengths and Limitations

Among the strengths of this study is the systematic comparison of multiple forms of early‐onset Non‐21OHD SW within a single cohort using uniform clinical, biochemical, and therapeutic variables, allowing direct comparison of early presentation, acute management, and early longitudinal course across etiologies. In addition, the availability of standardized follow‐up data enabled exploratory assessment of the relationship between initial presentation, treatment requirements, and auxological outcomes.

Several limitations must be acknowledged. The retrospective design and the small sample size, inherent to the rarity of these conditions, limited statistical power and precluded definitive inferential conclusions. As a result, all correlation analyses should be interpreted as exploratory. Documented molecular confirmation was not available in the retrieved records for some older cases included in this long‐term cohort. Although these patients were retained only when the recorded diagnosis was consistent with the available clinical, biochemical, hormonal, therapeutic, and longitudinal data, this remains a limitation of the retrospective design. Biochemical assessments at presentation were not always uniformly timed, reflecting real‐world emergency management but potentially limiting the precision of severity classification. Similarly, treatment‐related variables were collected retrospectively from routine clinical care and may have been influenced by differences in monitoring intensity, timing of biochemical reassessment, and dose‐adjustment practices. For this reason, treatment doses and time to electrolyte stabilization should be interpreted as descriptive indicators of real‐world management rather than as robust comparative endpoints across diagnostic groups. A further point concerns the biochemical definition of SW used in this study. Although serum sodium < 130 mEq/L was used to describe the acute SW episode, this threshold did not affect the composition of our cohort, since all patients retrospectively identified as having clinically diagnosed endocrine Non‐21OHD SW fulfilled this criterion at presentation. Nevertheless, we acknowledge that, in a broader diagnostic setting, reliance on a sodium‐based definition may fail to capture mild or evolving forms of mineralocorticoid deficiency or resistance, particularly ASD or PHA1 cases presenting initially with isolated or predominant hyperkalemia. Similarly, the 60‐day limit for clinical presentation was used to define an early‐onset cohort focused on neonatal and very‐early‐infancy SW presentations. Therefore, milder congenital endocrine disorders presenting later in infancy were outside the scope of this study. Finally, follow‐up duration remains relatively short for conditions with lifelong implications, and longer prospective studies are needed to better define long‐term outcomes and treatment trajectories.

## 5 Conclusions

Early‐onset Non‐21OHD SW disorders remain diagnostically challenging because substantial phenotypic overlap at presentation may limit early etiological distinction, while clinically meaningful differences emerge more clearly over time. In this setting, assessment during the acute presentation is mainly aimed at prompt stabilization, whereas the real clinical burden of each disorder becomes clearer during follow‐up through differences in treatment needs, diagnostic evolution, and short‐term clinical course. These observations highlight the importance of considering rarer endocrine causes of early‐onset SW, particularly in symptomatic infants with negative newborn screening for 21OHD CAH, and suggest that targeted molecular testing panels may help identify the underlying disorder when clinical and biochemical features overlap. Timely diagnostic clarification may support treatment planning, follow‐up, and counselling.

## Author Contributions

**Vittorio Ferrari:** conceptualization, investigation, writing – original draft, methodology, validation, visualization, writing – review and editing, formal analysis, data curation, software. **Emma Bonaguri:** conceptualization, validation, visualization, writing – review and editing, formal analysis, data curation. **Giovanni Silva:** conceptualization, validation, visualization, writing – review and editing, formal analysis, data curation. **Rita Ortolano:** conceptualization, investigation, writing – original draft, writing – review and editing, supervision, data curation, validation, methodology, software. **Marcello Lanari:** supervision, data curation, conceptualization, investigation, methodology, validation, writing – review and editing, resources, project administration. **Valentina Assirelli:** conceptualization, investigation, methodology, writing – review and editing, validation, data curation. **Antonio Balsamo:** conceptualization, investigation, methodology, validation, writing – review and editing, writing – original draft, formal analysis, resources, supervision, data curation. **Federico Baronio:** conceptualization, investigation, methodology, validation, writing – review and editing, supervision, data curation.

## Funding

The authors have nothing to report.

## Conflicts of Interest

The authors declare no conflicts of interest.

## Data Availability

The data that support the findings of this study are available on request from the corresponding author. The data are not publicly available due to privacy or ethical restrictions.
